# Are associations between socio-economic characteristics and exposure to air pollution a question of study area size? An example from Scania, Sweden

**DOI:** 10.1186/1476-072X-4-30

**Published:** 2005-11-16

**Authors:** Emilie Stroh, Anna Oudin, Susanna Gustafsson, Petter Pilesjö, Lars Harrie, Ulf Strömberg, Kristina Jakobsson

**Affiliations:** 1 GIS Centre & The Department of Physical Geography and Ecosystem Analysis, Lund University, Sölvegatan 12, SE-223 62 Lund, Sweden; 2 Department of Occupational and Environmental Medicine, Lund University Hospital, SE-221 85 Lund, Sweden

## Abstract

**Background:**

Numerous studies have shown that exposure to air pollutants in the area of residence and the socio-economic status of an individual may be related. Therefore, when conducting an epidemiological study on the health effect of air pollution, socio-economy may act as a confounding factor. In this paper we examine to what extent socio-economic status and concentrations of NO_2 _in the county/region of Scania, southern Sweden, are associated and if such associations between these factors differ when studying them at county or city level. To perform this study we used high-resolution census data and modelled the annual exposure to NO_2 _using an emission database, a dispersion modelling program and a geographical information system (GIS).

**Results:**

The results from this study confirm that socio-economic status and the levels of NO_2 _in the area of residence are associated in some cities. The associations vary considerably between cities within the same county (Scania). Even for cities of similar sizes and population bases the associations observed are different. Studying the cities together or separately yields contradictory results, especially when education is used as a socio-economic indicator.

**Conclusion:**

Four conclusions have been drawn from the results of this study. 1) Adjusting for socio-economy is important when investigating the health effects of air pollution. 2) The county of Scania seems to be heterogeneous regarding the association between air pollution and socio-economy. 3) The relationship between air pollution and socio-economy differs in the five cities included in our study, depending on whether they are analysed separately or together. It is therefore inadvisable to determine and analyse associations between socio-economy and exposure to air pollutants on county level. This study indicates that the size and choice of study area is of great importance. 4) The selection of socio-economic indices (in this study: *country of birth *and *education level*) is important.

## Background

Humans are inevitably, although we constantly seem to forget this, part of the environment we live in. The way we interact with and affect the environment will have consequences not only for our surroundings but also ultimately on ourselves. Air pollution is an example of an anthropogenic effect that has become one of the major health hazards of our time. Airborne pollutants generated by traffic, industry, energy consumption, combustion, etc. are believed to cause not only respiratory and cardiovascular diseases in those exposed to them, but they may also cause premature death in certain groups of vulnerable people. Exposure to different air pollutants and associations to various respiratory and cardiovascular diseases have been the subject of intense study during recent years [1-5]. It has been shown that short-term variations in air pollution levels are related to lung function and asthma symptoms [6], as well as mortality and hospital admissions due to cerebrovascular disease and heart disease [7]. Particular matter ≤ 10 μm (PM10) and ozone are usually considered to be the air pollutants having the greatest effects on health but NO_2 _is a good indicator of air pollutants in general [8], and we have thus chosen this component as the indicator of exposure element in this study.

There is an element of discrimination in the way we are exposed to air pollutants. The area affected and the people exposed may be miles away from the source of the pollutants, which makes it difficult not only to control and regulate for pollution, but also to analyse its effects on health. The effect of the pollution may also differ depending on weather conditions or, for example, the socio-economic characteristics of the people exposed. Another difficulty is that the health effects of air pollution are similar to those of many other diseases caused by social factors such as poor dietary habits, lack of exercise and stress. Since both air pollutants and socio-economic status strongly influence health [9-11] socio-economic factors can act as confounders when studying the health effects of air pollutants [6], making it difficult to quantify the health effects of air pollutants alone.

Numerous studies have shown that groups with low socio-economic status tend to live in areas that are exposed to air pollutants to a greater extent than groups with high socio-economic status [12-16]. Several studies on the relation between geographical location and health have been conducted during the past 30 years; many of them confirming that socio-economic status can act as a confounder when investigating the exposure to different health hazards in the area of residence. According to Bowen (2002), who has reviewed 42 empirical research studies on the subject, many of these studies are based on such poor empirical foundations that the results should be viewed as unreliable [17]. Bowen also states that many of these studies fail to detect the underlying spatial process involved due to badly chosen geographical units. Willis, Jerrett, Burnett and Krewski (2003) studied the impact of the size of the study area (metropolitan areas versus county areas) on the results of a study on the relationship between long-term exposure to sulphate air pollution and mortality [18]. Their results clearly shows that the size of the study area can have an impact on the results of an epidemiological study, but that there are both advantages and disadvantages of large and small study areas.

It is important to be able to describe socio-economy on a group level when evaluating possible contextual health effects. A contextual effect is an effect where the group influences the individual, for example, when the socio-economic characteristics of a neighbourhood influence the effect on the health of an individual caused by air pollution. In a multi-level model, where data are analysed according to an individual's group affiliation, contextual effects are of particular interest. Here, group affiliation is determined by area of residence. Individuals from the same group are assumed to both influence, and be influenced by, group membership. This assumption might not be valid if a group is very heterogeneous. In the future we intend to analyse health effects of air pollution in Scania with multi-level models, as the level of socio-economy within an area of residence might have a contextual influence. Clearly, the definition of the area can affect the level, as well as the heterogeneity of socio-economy within that area. In this work, we investigated the relationship between air pollution and socio-economy in Scania, in order to define relevant contextual areas for further studies.

Improved technologies and more detailed data sources have made it possible to model personal exposure to air pollutants and socio-economic status. This study analyses the association between socio-economic status and mean annual concentrations of NO_2 _in Scania, as well as the possible influence of the choice of geographical level and study area on the results.

**Figure 1:**
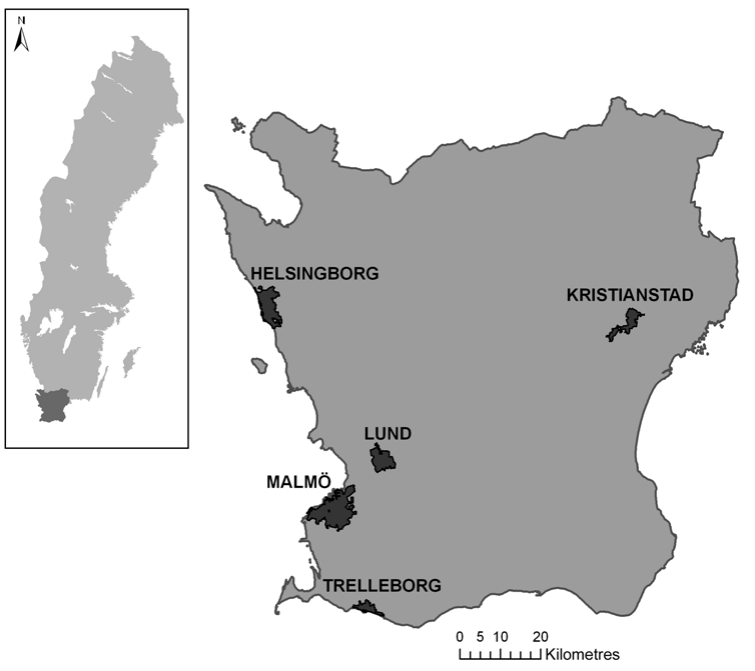
The county of Scania, Sweden, and five of the major cities in the region.

The present study was carried out on the population of the whole county of Scania in southern Sweden (described below) in 2001, separately for the five major cities in the region: Malmö, Helsingborg, Lund, Kristianstad and Trelleborg (Figure 1), and for the five cities grouped together in one data set. These cities differ in geographical location, infrastructure and population size, as well as in socio-economic structure. In order not to confound the analysis with a rural-urban gradient, the analyses of the five cities were performed strictly on residents within the city limits.

### Scania

Scania is the southernmost county in Sweden. It covers around 11,350 km^2^, which is approximately 2% of the total Swedish land area. The region is densely populated, with more than 1.1 million people living in the area, which is approximately 11% of the total Swedish population. In Scania, approximately 67% of the population lives along the west coast. Most road traffic (passenger cars as well as trucks) from the European continent to Sweden and Norway passes through this area, and five motorways run through the region. There are also several harbours in the region and a considerable amount of cargo shipping and ferry transport along the coast. These factors, and the closeness to Copenhagen in Denmark, and the European continent, contribute to high concentrations of air pollutants in the region, compared with most other regions in Sweden.

### Malmö

Malmö is the largest city in Scania, with a population of about 260,000 (the third largest city in Sweden) and an area of 66 km^2^. The city is the residential focus of the rich agricultural area of Scania, and used to be one of Sweden's most important industrial and trading cities. During the past 20 years industry and commerce in the area have decreased quite considerably, resulting in a sudden rise in the proportion of unemployed, to almost twice the national average [19]. During recent years, this number has begun to slowly decrease as the city has begun to adapt to new areas of the labour market. The completion of the Öresund bridge in 2000, connecting the mainland of Sweden with Denmark, has expanded the labour market for the residents in both Malmö and Copenhagen. The commuting between Sweden and Denmark now dominates commuter traffic, and this almost doubled between 2001 and 1997 [20,21]. Malmö is known for its high proportion of immigrants, with more than 20% of its residents born outside Sweden [22]. The city is considered to be one of Sweden's most segregated [23].

### Helsingborg

Helsingborg is the second largest city in Scania and covers approximately 35 km^2^. It has a population of 89,000. The city has one of the most important harbours in Sweden and is of great importance as a transportation link for trains. Both the ferry traffic and commercial traffic are of great importance to the city. The distance to the Danish mainland across the strait is only 6 km, and the ferry traffic between the two countries is intense. The city is considered to be segregated, with a north/south socio-economic gradient [24].

### Lund

Lund has a population of 76,000 residents and covers an area of 23 km^2^. The city is in many ways characterised by the university, and around one third of the residents are students. Thus the city's residents are younger than the national average, and especially the age group 20–29 is highly overrepresented. Due to the high number of students and young people, relative to the population size, migration is much higher than in the other cities. The university, the science park and the university teaching hospital are three of the major employers. The city is not as segregated as Malmö and Helsingborg which, in many ways, can be explained by the high migration to and from the city [25].

### Kristianstad

Kristianstad is situated on the east coast of Scania. It has a population of 32,000 and an area of 19 km^2^. The city is the centre of Scania's eastern region and is situated in one of Sweden's largest fruit-growing districts. Kristianstad is an active centre of commerce in the region and most of its residents are involved in the trade or service sectors. Although the city is not as segregated as Malmö and Helsingborg, there are still distinct differences in socio-economy between the city's neighbourhoods [26].

### Trelleborg

Trelleborg is the most southerly, as well as the smallest, of the cities included in the study. It has a population of only 24,000 inhabitants and an area of 10 km^2^. The city is highly affected by the busy trade and ferry traffic with the German harbours in Travemünde, Sassnitz and Rostock. Although the city is small, the fact that it is situated in a lively metropolitan area and the hectic trade traffic result in the same social problems as in the larger cities in the region. The proportion of residents with a high educational level is lower than the national average [27].

### Aims

The main aim of this study was to describe associations between the levels of mean annual concentrations of air pollution (NO_2_) and two socio-economic indices ("country of birth" and "level of education") in the region of Scania in Sweden. These associations are of special interest for future studies of health effects resulting from exposure to air pollutants, since socio-economy might act as a confounding factor, both individually and contextually in such a study.

A secondary aim of this study was to investigate the possible influence of differences in size (or level) of the study area on any associations observed. Do the size and choice of the study area affect the associations seen, and if so, to what extent? The answer to this question is of utmost importance, not only for this study, but for health effect studies in general. It is important to establish the effects of socio-economy to ensure that the results of epidemiological studies in general are not biased. If the associations observed are dependent on the size and choice of the study area, generalising the contextual effect of socio-economy for too large an area may lead to erroneous results.

## Results

**Figure 2:**
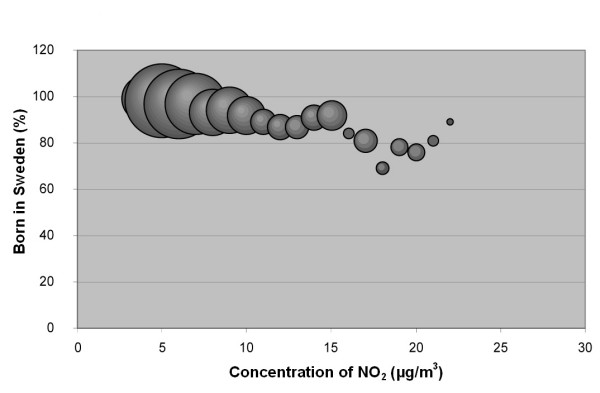
**Bubble diagram for Scania; Percentage of population in Scania born in Sweden versus annual mean concentration of NO_2 _at their real estate**. The bubbles are sized according to the weight of the estimate, which depends on the size of the population that the estimate is based on. The weight is thus a measure of the uncertainty in the estimate, and large bubbles represent more certain and influential estimates than small bubbles.

**Figure 3:**
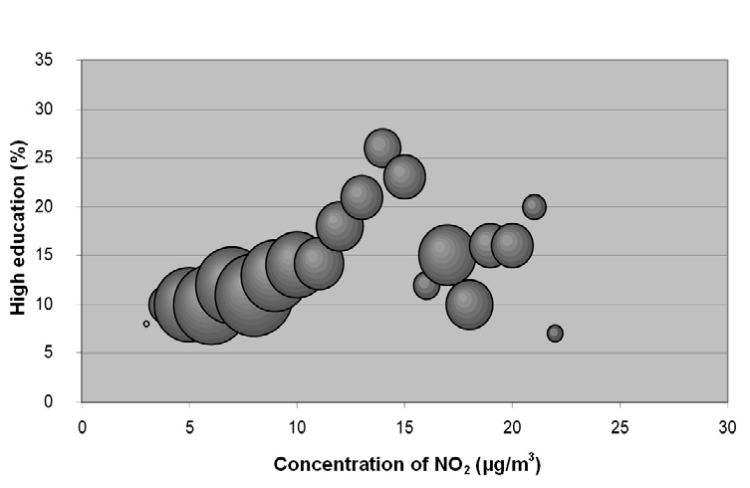
**Bubble diagram for Scania; Percentage of population in Scania with high education versus annual mean concentration of NO_2 _at their real estate**. The bubbles are sized according to the weight of the estimate, which depends on the size of the population that the estimate is based on. The weight is thus a measure of the uncertainty in the estimate, and large bubbles represent more certain and influential estimates than small bubbles.

**Figure 4:**
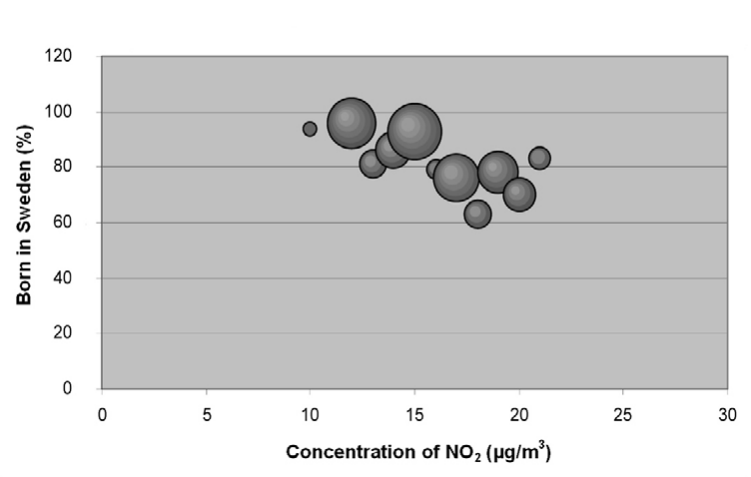
**Bubble diagram for Malmö; Percentage of population in Malmö born in Sweden versus annual mean concentration of NO_2 _at their real estate**. The bubbles are sized according to the weight of the estimate, which depends on the size of the population that the estimate is based on. The weight is thus a measure of the uncertainty in the estimate, and large bubbles represent more certain and influential estimates than small bubbles.

**Figure 5:**
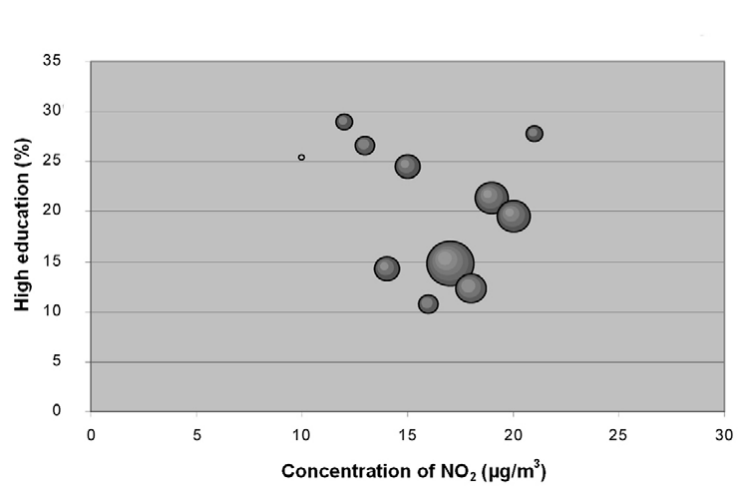
**Bubble diagram for Malmö; Percentage of population in Malmö with high education versus annual mean concentration of NO_2 _at their real estate**. The bubbles are sized according to the weight of the estimate, which depends on the size of the population that the estimate is based on. The weight is thus a measure of the uncertainty in the estimate, and large bubbles represent more certain and influential estimates than small bubbles.

**Figure 6:**
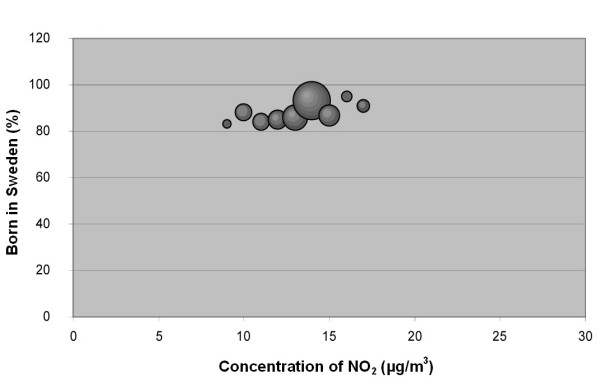
**Bubble diagram for Lund; Percentage of population in Lund born in Sweden versus annual mean concentration of NO_2 _at their real estate**. The bubbles are sized according to the weight of the estimate, which depends on the size of the population that the estimate is based on. The weight is thus a measure of the uncertainty in the estimate, and large bubbles represent more certain and influential estimates than small bubbles.

**Figure 7:**
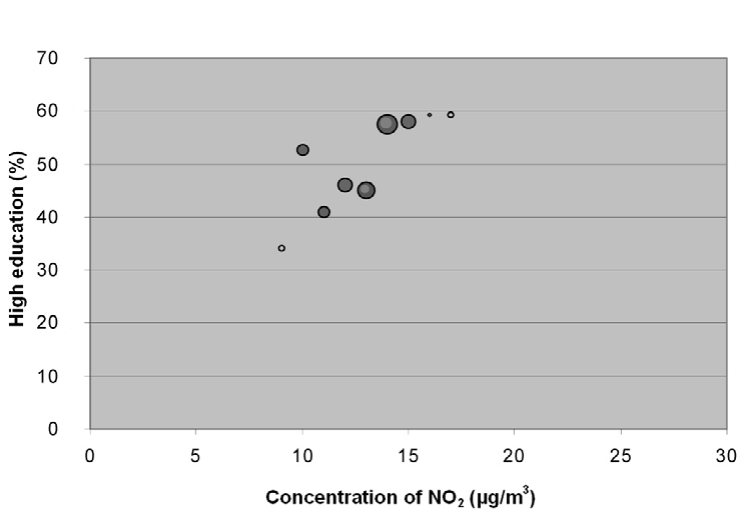
**Bubble diagram for Lund; Percentage of population in Lund with high education versus annual mean concentration of NO_2 _at their real estate**. The bubbles are sized according to the weight of the estimate, which depends on the size of the population that the estimate is based on. The weight is thus a measure of the uncertainty in the estimate, and large bubbles represent more certain and influential estimates than small bubbles.

The results of this study confirm the hypothesis that associations exist between socio-economic status and NO_2 _concentration in our study area (Table 1,2 and Figure 2, 3, 4, 5, 6, 7). According to the Spearman correlation analysis there is a statistically significant correlation between both country of birth and the level of education, not only in Scania as a whole, but also in all five cities included in the study, regardless of whether they are studied separately or together. The correlation coefficients are often low, however.

**Table 1:** Descriptive statistics for the population in Scania, Sweden. Statistics for the population in Scania, Sweden (2001) and the five cities in the region (Malmö, Helsingborg, Lund, Kristianstad and Trelleborg). Statistics for the socio-economic subgroup relating to educational level were calculated for the age group 25–64 years of age, while the subgroup "country of birth" includes all the habitants of the region/city.

**Inhabitants**	**Scania**	**Malmö**	**Helsingborg**	**Lund**	**Kristianstad**	**Trelleborg**
Number of inhabitants	1,165,411	258,140	88,992	76,334	32,295	24,254
Born in Sweden (%)	87	75	83	83	86	84
Born in OC (%)	10	20	12	12	12	11
Low education (%)	82	81	84	49	82	92
High education (%)	18	19	16	51	18	8

**Concentrations of NO_2 _(μg/m^3^)**						

Median level	14	17	13	13	9	12
Quartiles	9/17	15/19	12/20	12/14	8/10	11/18
Min	3	10	10	9	6	7
Max	23	23	21	17	11	22

**Table 2:** Correlation between socio-economic subgroups and mean annual exposure to NO_2 _in Scania, Sweden (2001). The correlation coefficients for those born in OC (Other Country) and those with a low level of education are not presented since they are the exact inverse of the correlation coefficients for those born in Sweden, and those who are highly educated, respectively. The proportion with:"high level of education and concentration of NO_2_" is calculated for the age-group 25–64 years.

**Spearman's correlation coefficient and p-value**			
**Area**		**Proportion born in Sweden and concentration of NO_2_**	**Proportion with high level of education and concentration of NO_2_**

**Scania**	Correlation coefficient	-0.9	0.2
	p-value	<0.001	<0.001
**All 5 cities together**	Correlation coefficient	-0.5	-0.4
	p-value	<0.001	<0.001
**Malmö**	Correlation coefficient	-0.8	0.1
	p-value	<0.001	<0.001
**Helsingborg**	Correlation coefficient	0.1	0.2
	p-value	<0.001	<0.001
**Lund**	Correlation coefficient	0.6	0.8
	p-value	<0.001	<0.001
**Kristianstad**	Correlation coefficient	-0.3	0.2
	p-value	<0.001	<0.001
**Trelleborg**	Correlation coefficient	0.1	0.5
	p-value	<0.001	<0.001

The associations seen are not consistent between cities. Also, the sign of the associations differs between the two socio-economic indexes, i.e. "country of birth" and "level of education" show opposite correlations to the level of pollution.

The stronger correlation coefficients in the study implied that associations exists between country of birth and NO_2 _in Scania (negative), in all cities together (negative), in Malmö (negative), and in Lund (positive). The association between country of birth and level of NO_2 _implies that being an immigrant in Scania, Malmö or the five study cities (analysed as a group) is associated with elevated levels of NO_2_. In Lund, however, the association between NO_2 _concentrations and country of birth is the opposite, implying that being born in Sweden is associated with higher levels of NO_2_.

A correlation was observed between education level and concentration of NO_2 _in the cities together (negative), in Lund (positive) and Trelleborg (positive). This implies that less educated groups of people, living in the five cities studied and in Scania in general, are exposed to higher levels of NO_2_. This is, however, not the case in Lund and Trelleborg when studied separately, where highly educated residents seemed to be exposed to higher concentrations of NO_2_. We found that the sign of the correlation coefficient for education was positive in all cities when studied separately, but negative when analysed as one data set.

## Discussion

This study confirms that associations exist between socio-economy and level of air pollution (Table 2). It is important to distinguish between a low p-value and a strong correlation. A low p-value does not imply that the association is strong, only that the association observed is not likely to occur due to chance, regardless of how weak the association actually is. The p-values in this study are all < 0.001 as a result of the many observations, while many of the correlation coefficients are below 0.5, which can be considered as indicating weak associations.

The results are not consistent; neither between cities nor between the socio-economic indexes, nor when analysing all cities together or separately. The consistency of socio-economic indexes will not be discussed further here, but we conclude that it appears important to gather as much information as possible about socio-economy to describe socio-economic status.

The results obtained when analysing the cities together in one data set were not at all consistent with the results when considering them separately, especially regarding level of education, where even the sign of the association differed between the combined data set and the separate cities (Table 2). Therefore, generalizing associations between socio-economy and air pollution from a regional level to a city level can give erroneous results. Although there seems to be a strong correlation between NO_2 _concentration and country of birth (Figure 2) this correspondence is probably strongly influenced by a rural-urban gradient, resulting in a biased relation between this socio-economic variable and the exposure level. Most immigrants in Sweden tend to move to cities, especially larger cities, rather than settle in the countryside [28]. Since the levels of NO_2 _are significantly lower outside cities, this gradient probably makes a major contribution to the strong correlation observed between the concentration of NO_2 _and "country of birth" in the whole county of Scania. On the regional level there may also be a risk of relations cancelling each other out, since they are not consistent between cities.

In Malmö, the negative correlation for the group born in Sweden implies that this group tends to live in areas with lower concentrations of NO_2 _than the group born in other countries (Figure 4). The proportion of immigrants in the different areas of Malmö is uneven which makes Malmö a segregated city. The neighbourhoods with high proportions of immigrants are located in the outskirts of the city. Since Malmö is surrounded by a ring road at which three of the five main motorways in the area converge, the emissions in these neighbourhoods tend to be higher than in other parts of Malmö.

In Helsingborg the correlation coefficients shows no correlation at all between the socio-economic variables analysed and levels of NO_2_. This is probably due to differences in directions between the socio-economic gradients and the concentration gradient for NO_2_. Helsingborg stretches along the coast of the strait between Sweden and Denmark, making the city quite long and narrow in a north/south direction, with a distinct socio-economic gradient in the same direction. The major source of air pollutants in the area is the lively ferry traffic along and across the strait, as well as from the three motorways that converge and run alongside the city. These factors cause the city to have the same variation in NO_2 _levels in the north south direction but vary in the west easter direction. This results in evening out of the exposure to air pollutants between the different socio-economic groups.

In Lund the correlation is positive for both the socio-economic variables, implying that people born in Sweden and individuals with a high level of education tend to live in areas with higher levels of NO_2 _than people born outside Sweden or with lower levels of education (Figure 6 and 7). This is probably the result of a phenomenon observed in many metropolitan areas, i.e. it is very desirable to live in the city centre where the cost of accommodation is high, and can usually only be afforded by people with high economic status. Since pollution levels are higher in the city centre these people tend to be more highly exposed. It is worth noting that this phenomenon is probably present in Malmö as well, although it does not compensate for the other socio-economic and NO_2 _gradients mentioned above.

In Kristianstad, only weak associations were seen between socio-economy and air pollution. Compared with the other cities in the study, the levels of NO_2 _in Kristianstad are, much lower, and less spread (Table 1). The small range in exposure decreases the possibilities of detecting an association

The positive correlation between the level of education and the exposure to NO_2 _seen in Trelleborg is weaker but implies a similar explanation to that in Lund, where highly educated people tend to live in the more central areas of the city. The fact that Trelleborg is located along the coast with a large, busy industrial harbour in the middle of the city also increases the risk of people living in the city centre of being highly exposed to NO_2 _and other air pollutants derived from the ferry traffic.

As already mentioned, the socio-economic indexes country of birth and educational level do not show the same associations to concentration of NO_2 _in our study. Strong associations are seen between country of birth and concentration of NO_2 _and regarding education and concentration of NO_2_, but not in the same cities (with Lund as an exception). The analysis regarding education was performed on the population between 25 and 64 years old only, while the analysis regarding country of birth was performed on the whole population. In order to investigate whether the difference in results between the two socio-economic indexes is a result of different age distributions, the analysis of the association between country of birth and concentration of NO_2 _was also performed on the age group 25–64 years. This did not alter the results. Thus the different age distributions do not explain why "country of birth" and "level of education" do not show the same associations with concentration of NO_2_.

We chose to analyse the data with simple correlation analysis. Geographical Weighted Regression (GWR) models provide a more sophisticated analysis, but such models are not suited to our purpose. GWR analyses spatially varying relationships by weighting them to form one surface for each relationship studied. Since one of the aims of our study was to investigate socio-economic associations with air pollution in separate cities, GWR is not suitable.

Valid exposure assignments are naturally of particular importance in reducing bias. Despite the fact that this study does not focus on this problem, we can not stress enough the importance of using a functional dispersion models and valid exposure data for exposure/concentration response relationship studies.

The inconsistency in the relationship between socio-economy and NO_2 _between cities, as well as over larger areas, may conceal existing associations [29]. Performing an analysis for a large area and then generalising the results to the whole country or region is not recommended since the socio-economic factors in larger cities in the area or the rural-urban gradient might dominate the results.

The results of this study clearly show that the size and type of the study area have an impact on the findings when investigating possible associations between socio-economy and exposure to air pollution. Relations observed on one area level may be almost the opposite on a larger scale. This is the case for the associations seen between country of birth and exposure to NO_2 _for the residents of Lund, where the correlation coefficient implies that being born in Sweden is associated with high levels of NO_2_. When Lund is included in the group of five cities studied the opposite association would, however, be implied namely that being an immigrant and living in any of these five cities would increase the risk of being highly exposed to NO_2_. This would be even more misleading if these cities were to be included in a regional study of the whole area of Scania. The smallest area studied was a city. It would have been interesting to divide the data further into districts, to see how such a division influenced the relationships.

## Conclusion

The results of this study show a covariation between socio-economic status and the levels of NO_2 _in the area of residence in Scania, and thereby confirm that socio-economy could act as a confounding factor and create bias when investigating health effects of air pollution. In order to quantify the magnitude of bias, several other assumptions have to be made, such as risk levels for various groups of air pollution, smoking habits and other important variables (age, sex, etc).

In this study the exposure to air pollutants of different socio-economic groups varies considerably between areas. It has been shown that the relationships can differ, not only in magnitude but also in direction, whether splitting the study area into small areas or not, as seen for level of education. Relations can be the opposite in cities with similar sizes and populations. Larger areas, such as the county of Scania, are influenced by a large number of socio-economic gradients. The urban-rural gradient makes it especially difficult to determine and analyse associations between socio-economic variables and exposure to air pollutants on a regional level.

In epidemiological studies on the health effects of air pollution, incorporation of individual-level and area-level data on various socio-economic variables (i.e. country of birth, educational level, etc.) is important. Our results demonstrate that there is no consistency in how individuals in different socio-economic classes are exposed to air pollutants over a larger area, and that both the size of the area, and the choice of socio-economic indexes affect the associations observed.

## Methods

The procedure used to analyse the covariation between socio-economic factors and exposure to air pollutants is described below:

1. Modelling the concentration of NO_2_

An emission database and a GIS program were used to model the levels of NO_2 _in Scania. This was done using a dispersion modelling program and emission data for the year 2001 together with meteorological data, resulting in a grid giving mean annual concentrations of NO_2 _for Scania in 2001.

2. Modelling the exposure to NO_2_

Mean annual NO_2 _exposure to each individual was modelled in a GIS program using the mean annual concentrations of NO_2 _and population data sets.

3. Division of the population into socio-economic groups

The population data were divided into socio-economic groups using the indexes *country of birth *and *level of education*.

4. Statistical analysis

The data were statistically analysed depending on each individual's socio-economic status and exposure to NO_2 _using a weighted Spearman rank correlation.

### Modelling the concentration of NO_2_

To model the concentrations of NO_2 _for Scania an NO_x _emission database with approximately 24,000 sources for the area was used [30]. The different sources of emissions consist of roughly 23,500 line sources corresponding to the major roads and ship routes, 500 point sources corresponding to most of the larger industrial plants and approximately 50 area sources corresponding to emissions from heating, wood burning, farming machinery, construction machinery, etc. The emission database includes time variations for different pollutants depending on hour, day and month of the year. The emission database does not include any continental contribution, i.e. sources outside Sweden.

The software Enviman was used to model the concentrations of NO_x _(later converted into NO_2 _levels). Enviman uses a Gaussian model: AERMOD, a USEPA model [31]. The meteorological parameters in the calculation were based on climatological series for the period 1995–2003 gathered from a meteorological weather station in central Malmö [32].

**Figure 8:**
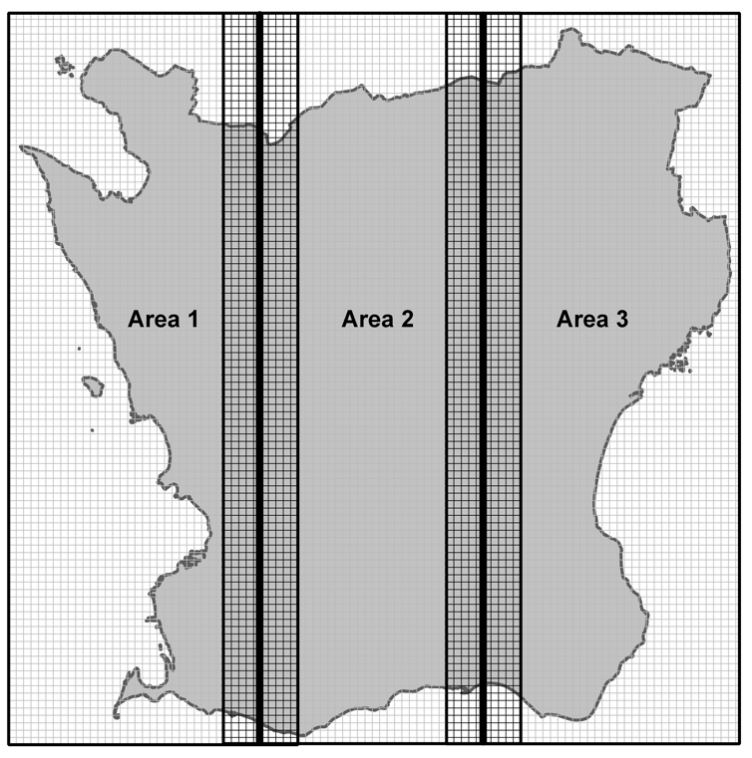
**Creating a continuous concentration grid of NO_2 _for Scania**. The mean annual concentrations of NO_2 _in μg/m^3 ^for Scania were modelled in three separate areas (1–3) each overlapping its neighbouring areas by 10 kilometres to prevent modelling errors in the border areas. These overlaps were later removed and the areas merged into a continuous concentration grid.

In this study, the mean annual value of NO_x _was modelled with a spatial resolution of 250 × 250 m for the county of Scania. Both the levels and the spatial variation of the modelled concentrations of air pollutants are of interest. Therefore, the geographic resolution of the modelling grid is of vital importance. If the resolution is too low the model might generate a grid with lower variations in concentration between the cells. This might increase the uncertainty in the following exposure estimates. Modelling with higher resolution increases the opportunity to detect variations in space as well as extreme values. The capacity of the modelling tool and the computer, as well as the resolution and quality of the data in the emission database, set the limit for the highest resolution that is reasonable to model. To be able to model with this relatively high resolution the county was divided into three parts by splitting the region into three equally sized areas stretching from north to south. Each of these three areas had a 10-kilometre overlap with their neighbouring areas to prevent modelling errors that might otherwise appear along the borders (Figure 8). For each of the three areas two modelling operations were carried out: one to modelled the concentrations from local sources within that specific area, and a second to model the regional contribution of the NOx concentrations in each area contributed by the two neighbouring areas in the region. The local and regional concentrations for each cell were summed, and removing the 10-kilometre overlap between the areas and merging them together created a continuous raster.

To convert the concentration of NO_x _to NO_2 _the following equation were used [32]:

*NO*_*x *_≤ 13.85 *μg*/*m*^3 ^⇒ *NO*_2 _=*NO*_*x *_

*NO*_*x *_> 13.85 *μg*/*m*^3 ^⇒ *NO*_2 _=8.8·()

To take the continental contribution into account, 2.5 μg/m^3 ^NO_2 _was added to the modelled concentration. The continental contribution was calculated as a regional mean from measurements from ten different reference stations in the area during the period 2000–2002 [30].

**Figure 9:**
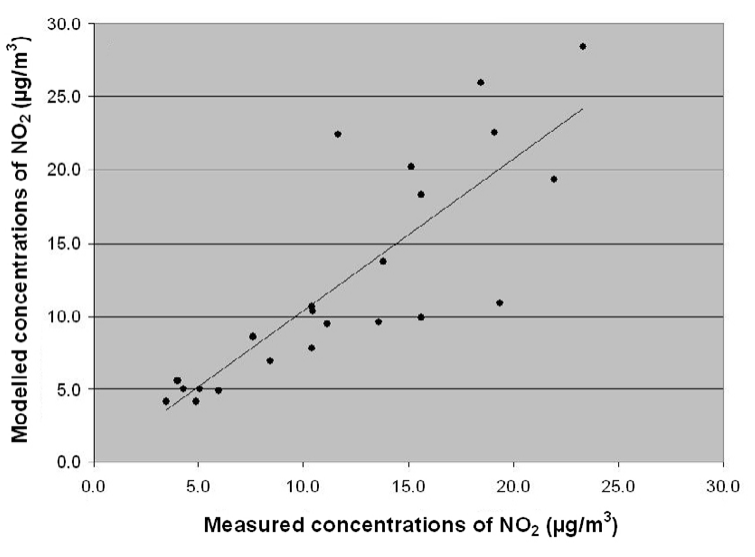
**Validation of the concentration model**. Modelled annual average concentration of NO_2 _(μg/m^3^) versus measured. The modelled concentration is plotted against measured concentration of the annual mean of NO_2 _from 23 measuring stations in Scania. y = 1.041x - 0.0605 R^2 ^= 0.69

Finally, the concentration modelling was validated with measurements from 23 measuring stations in Scania. The yearly means for these were compared with the modelled annual concentrations of NO_2 _for the year 2001. The validation of the concentration modelling (Figure 9) gave a correlation coefficient of 0.69. For concentrations up to 10 μg/m^3 ^the modelled concentrations are quite close to the measured values, while at concentrations above 10 μg/m^3 ^the model seems to underestimate concentrations ranking from 10 to 15 μg/m^3 ^and overestimate concentrations ranking from 15 to 25 μg/m^3^.

**Figure 10:**
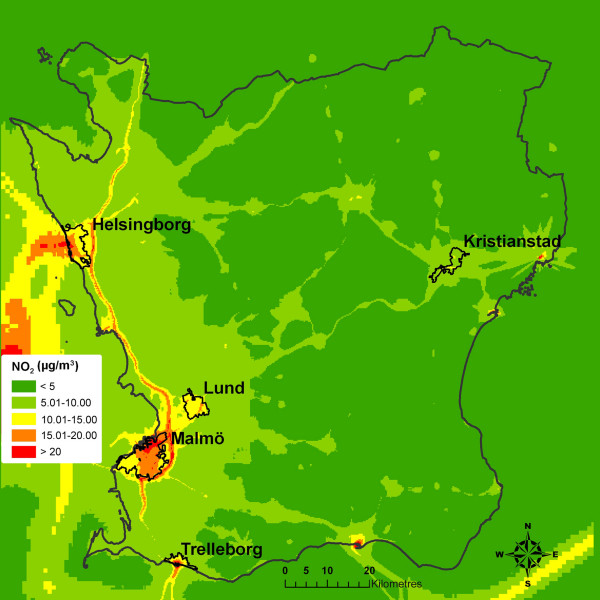
Modelled annual mean concentrations of NO_2 _for Scania (2001).

The concentration modelling (Figure 10) shows that there are considerably higher levels of NO_2 _in the western regions than in the less populated eastern part of the county. The emissions of NO_2 _from the three major cities in the region (Malmö, Helsingborg and Lund) can be seen clearly, as well as the major roads in the county. Also, the ferry traffic along the coast and some of the contributing emissions from Denmark in the west are visible. Areas with NO_2 _levels lower than 5 μg/m^3 ^have a very low population density and consist mainly of agricultural land or forests.

### Modelling the exposure to NO_2_

In this study, two different sets of population data were used (henceforth referred to as PD1 and PD2). Both of the data sets were obtained from the Regional Office of Scania, Sweden, and are based on the Swedish National Registry. The data sets are on individual level for the registered population in Scania during 2001, but they differ in spatial resolution and the amount of attribute data linked to them.

1. PD1

In PD1 individuals' location are represented by points at the centre coordinates of their real estate (listed in the National Registry). The only attributes linked to this data set were sex and age.

2. PD2

In PD2 the location of each individual's real estate is given as a centroid in a 1 km grid. PD2 was linked with socio-economic attributes from Statistics Sweden and contains the following attributes: sex, year of birth, country of birth, marital status, income for the previous year (2000) and highest educational level.

The task is to add exposure values to and all individuals in PD2. Since we cannot distinguish between the persons living in a 1 × 1 km cell all of them will get the same exposure value. This could be performed by only using only the information in PD2. However, the exposure value can be improved by using the detailed positions of the individuals in PD1 using the methodology described below.

**Figure 11:**
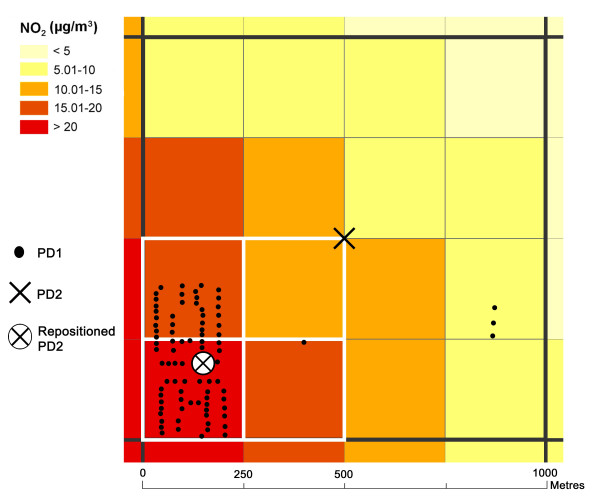
**Calculation of the geographical centre of the residents' real estates within each 1 × 1 kilometre grid cell**. Small grid cells: grid cells for modelled annual mean of NO_2 _(250 × 250 m). Large bold grid cell: 1 km^2^. Small black dots: the centre coordinates of the real estate (according to the National Registry) for each individual in population data set PD1. Black centre cross: the generalised position of the individual's real estate, within the specific km^2^, according to population data set PD2. White crossed circle: calculated geographical centre of the residents' real estate within the specific km^2^. White-edged grid cells: the cells within the concentration grid which, through bilinear interpolation were used to assign an NO_2 _exposure value to the individuals in the repositioned PD2.

The annual mean of NO_2 _in Scania for 2001 was modelled with a 250 × 250 m grid resolution (small coloured grid in Figure 11). Since the residences of the individuals within each square kilometre are not evenly distributed in space, it was decided to relocate the centre coordinate to a position within each square kilometre which better corresponded to the total population density within that specific square kilometre.

To find the geographical centre of the individuals' residences in PD2 the data set was combined with PD1. PD1 contained the exact location of each individual's real estate (small black dots in Figure 11). By creating a grid (large bold grid cell in Figure 11) out of the kilometre points of census data obtained in PD2 and applying a ID number to each of these cells (consisting of each specific cells X and Y centre coordinates) this ID number could then be transferred to the points in PD1, depending on within which square kilometre grid cell the points in PD1 were positioned.

For each set of points with the same ID number (i.e. those that fell within the same square kilometre grid cell) in PD1, the geographical centre was then estimated by calculating the average X and Y coordinate for all the points. The coordinates for this new centre point were then transferred back to PD2 and the individuals within this data set were subsequently repositioned from their centroids to these new coordinates (white crossed circle in Figure 11).

An approximation of the individual's average annual exposure to NO_2 _in μg/m^3 ^for these new locations was assigned to each individual in the repositioned population data set, PD2, by using bilinear interpolation (white edged grid cells in Figure 11) [33]. This relocation also increases the accuracy in the estimation of the NO_2 _concentration to which most of the individuals within each square kilometre were exposed [34].

### Division of the population into socio-economic groups

From the repositioned population set, PD2, which was linked with socio-economic attributes and NO_2 _concentrations, different subsets were created based on the individual's country of birth and their educational level.

#### Country of birth

In the field of environmental justice it is common to study ethnicity and exposure to various pollutants. Sweden is not as segregated as some countries regarding race, and therefore race as a measure of socio-economic status is not applicable here. Nevertheless, segregation between immigrants, as a mixed ethnic group, and native Swedes exists to various extents. In 2000 around 11% of the Swedish population had been born outside the country, and most of these immigrants were concentrated in metropolitan areas or larger cities [35].

Since immigrants from other countries tend to have difficulties getting into the labour market they often belong to the lower social and income groups. Therefore, depending on country of birth, the individuals were classified into two different groups: Sweden – individuals born in Sweden (1,007,958 individuals) OC: Other Countries (110,214 individuals) The OC group includes individuals from countries outside the Scandinavia, countries and the European Union in 2001 and those not from: Australia, Canada, Japan, New Zealand or the USA. Individuals who were born in Scandinavian countries other than Sweden (2% of the total population in Scania), countries belonging to the European Union in 2001 (1% of the total population in Scania) and the major economies: Australia, Canada, Japan, New Zealand and the USA (0.2% of the total population in Scania) were excluded from the analysis. This was done as individuals from these countries might immigrate to Sweden under different conditions and for different reasons than immigrants from the other countries.

In a preliminary study it was examined whether or not this exclusion would affect the results of the study. It was found that leaving out this group did not significantly affect the results of the study, due to the small number of individuals in this group, compared to the other two categories,

#### Level of education

The Swedish educational system has changed over the years, and the definition of an individual with a high educational level has varied considerably. To reduce birth-cohort bias and to focus on the working population the analysis of this index was only carried out on individuals in the age group 25–64 years. Information on the highest level of education was missing for some individuals. In total around 70% of the total population of Scania was included in this analysis.

The attributes regarding education were divided into seven different categories:

1. Pre-secondary education for less than nine years (9% of the population)

2. Pre-secondary education for nine years (11% of the population)

3. Secondary education for maximum two years (19% of the population)

4. Secondary education for three years or longer (13% of the population)

5. Post-secondary education for less than three years (9% of the population)

6. Post-secondary education for three years or longer (9% of the)

7. Postgraduate studies (0.6 % of the population)

These seven different classes were grouped into two generalised classes representing individuals with a high level education (groups 6 and 7) and those with a low level of education (groups 1 to 5).

### Statistical analysis

The data were analysed by calculating the Spearman rank correlation coefficients for the exposure to NO_2 _and the proportion of the population being highly educated and born in Sweden. A correlation coefficient is a number between -1 and 1 which measures the degree to which two variables are related. Spearman's correlation is based on ranks, and can be used to describe non-linear relationships. The levels of NO_2 _were rounded off to single-decimal numbers. The correlation coefficients were determined calculating the proportion with high education and the proportion born in Sweden for each NO_2 _level. The correlation coefficients for the association between level of NO_2 _and proportion being highly educated or born in Sweden were then calculated. Consequently, a positive correlation coefficient here shows that the level of NO_2 _increases with an increasing proportion of the population having a high socioeconomic status. Correlation coefficients in the range of -0.4 to 0.4 were regarded as weak correlations of minor importance.

The data were plotted in bubble diagrams, with the level of NO_2 _on the x-axis and the proportion belonging to each socio-economic group on the y-axis. The size of the bubbles represents the inverse variance of the proportion estimates, and thus represents the certainty in the estimate. A large bubble represents a proportion estimate that was calculated based on a larger group of people than a small bubble. The weights, *w*_*i*_, for NO_2 _concentration strata *i *was calculated according to:





where  is the estimated population proportion and *n*_*i *_is the number of people in stratum *i*.

Table 1 presents the size of the population, the proportion belonging to the socio-economic subgroups, the median, range, and quartiles of the concentration of NO_2 _in the population for the whole of Scania and for each city in the study.

## Authors' contributions

PP, KJ and US conceived the study and participated in its design and coordination.

US assisted with the statistical analysis.

LH helped to draft the manuscript.

SG carried out the NO_2 _concentration modelling.

AO performed the statistical analysis, the alignment and wrote part of the paper.

ES carried out the NO_2_ exposure modelling and wrote the final version of the paper.
